# Impact of measurement location on direct mitral regurgitation quantification using four-dimensional flow cardiovascular magnetic resonance

**DOI:** 10.1016/j.jocmr.2025.101847

**Published:** 2025-01-26

**Authors:** Adarsh Aratikatla, Taimur Safder, Gloria Ayuba, Vinesh Appadurai, Aakash Gupta, Michael Markl, James Thomas, Jeesoo Lee

**Affiliations:** a School of Medicine, The Royal College of Surgeons in Ireland, Dublin, Ireland; b Division of Cardiology, Feinberg School of Medicine, Northwestern University, Chicago, Illinois, USA; c Department of Radiology, Feinberg School of Medicine, Northwestern University, Chicago, Illinois, USA; d Department of Biomedical Engineering, McCormick School of Engineering, Northwestern University, Evanston, Illinois, USA

**Keywords:** Valvular regurgitation, Mitral regurgitation, Mitral valve disease, 4D flow CMR, 4D flow MRI

## Abstract

**Background:**

Four-dimensional (4D) flow cardiovascular magnetic resonance (CMR) shows promise for quantifying mitral regurgitation (MR) by allowing for direct regurgitant volume (RVol) measurement using a plane precisely placed at the MR jet. However, the ideal location of a measurement plane remains unclear. This study aims to systematically examine how varying measurement locations affect RVol quantification and determine the optimal location using the momentum conservation principle of a free jet.

**Methods:**

Patients diagnosed with MR by transthoracic echocardiography (TTE) and scheduled for CMR were prospectively recruited. Regurgitant jet flow volume (RVol_jet_) and regurgitant jet flow momentum (RMom_jet_) were quantified using 4D flow CMR at seven locations along the jet axis, x. The reference plane (mid-plane, x = 0 mm) was positioned at the peak velocity of the jet at each cardiac phase, and three additional planes were positioned on either side of the jet, each 2.5 mm apart. RVol_jet_ was compared to RVol_TTE_, measured by the proximal isovelocity surface area method, and RVol_indirect_, measured by subtracting aortic forward flow volume from the left ventricle stroke volume derived from two-dimensional phase contrast at the aortic valve and a stack of short-axis cine CMR techniques.

**Results:**

RVol_jet_ and RMom_jet_ were quantified in 45 patients (age 63±13, male 26). In patients with RVol_jet_ at x = 0 mm ≥ 10 mL (n = 25), RVol_jet_ consistently increased as the plane moved downstream. RVol_jet_ measured furthest upstream (x = −7.5 mm) was significantly lower (39±11%, p<0.001) and RVol_jet_ measured furthest downstream (x = 7.5 mm) was significantly higher (16±19%, p<0.001) than RVol_jet_ at x = 0 mm. RMom_jet_ similarly increased from x = −7.5 to 0 mm (57±12%, p<0.001) but stabilized from x = 0–7.5 mm (−2±17%). From x = −7.5 to 7.5 mm, RVol_jet_ was in consistent moderate agreement with RVol_indirect_ (n = 41, bias = −2±24 to 8±32 mL, intraclass correlation coefficient = 0.55–0.63, p<0.001).

**Conclusion:**

The location of a measurement plane significantly influences RVol quantification using the direct 4D flow CMR approach. Based on the converging profile of RMom_jet_, we propose the peak velocity of the jet as the optimal position.

## Graphical abstract

**Figure:**
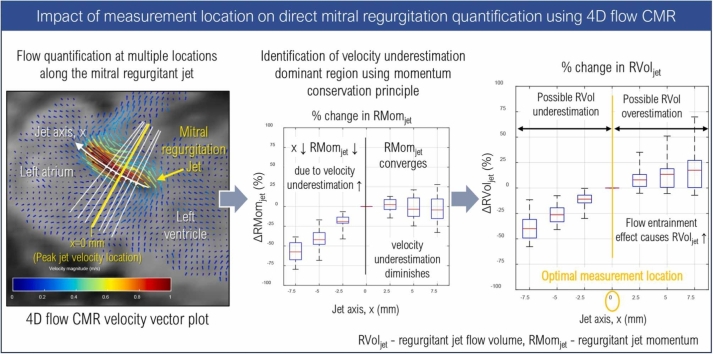

## 1 Introduction

Mitral regurgitation (MR) is the most common valvular heart defect, affecting 2% of adults in the United States, and has a prevalence increasing with age [1], [2], [3]. The risk of heart failure and death increases with worsening of MR severity [4]. Accurate quantification of MR severity is, therefore, of paramount importance for guiding appropriate patient management. Transthoracic echocardiography (TTE) serves as a clinical standard imaging modality to assess MR severity. TTE provides a comprehensive evaluation of structural and hemodynamic abnormalities associated with MR. However, accurate quantification of MR severity using TTE is hindered by its two-dimensional (2D) and one-directional velocity imaging constraints, necessitating simplification of complex morphologies of the regurgitant orifice and flow [5], [6], [7], [8], [9].

Cardiovascular magnetic resonance (CMR) is increasingly being used to assess MR with the advantage of offering reproducible quantification of regurgitant volume (RVol) [10], [11]. The most widely utilized method is an indirect volumetric approach by subtracting aortic forward flow volume from the left ventricle (LV) stroke volume measured using 2D phase-contrast images at the aortic valve and a stack of short-axis cine images [12]. While this indirect volumetric approach circumvents the issues of dealing with complex regurgitant orifice and flow, intrinsic errors in each measured volume are compounded due to subtraction. The presence of structural abnormalities, for example, interventricular shunt, mitral valve prolapse, and hypertrophic cardiomyopathy, can further compromise the accuracy of this method [13], [14], [15].

Four-dimensional (4D) flow CMR is a promising technique for overcoming limitations in conventional TTE- and CMR-based MR quantifications. 4D flow CMR captures three-directional velocity vectors within a volumetric region of the heart throughout the cardiac cycle. This enables retrospective placement of a flow measurement plane directly at the MR jet, while also adapting to the mitral valve movement [16], [17], [18], [19], [20], [21]. This direct quantification is thus relatively independent of complex morphology, movement, and pathology of the mitral valve and cardiac structures.

However, as the measurement plane can be placed anywhere along the jet, the “right” location to accurately estimate RVol remains unclear due to the absence of a gold-standard quantification technique. A commonly adopted strategy involves placing a plane slightly distal (typically 1–2 cm) to the mitral regurgitant orifice to avoid regions affected by severe signal loss near the orifice, associated with underestimation of velocity [17], [18], [19], [20], [21]. On the other hand, a prior in-vitro investigation suggests that as the measurement plane moves further away from the regurgitant orifice, RVol can be increasingly overestimated due to the flow entrainment effect [22]. The flow entrainment effect refers to a fluid dynamic phenomenon where a fluid jet draws surrounding fluid into itself as it propagates, resulting in a continuous increase in the jet flow rate [23]. The variability in RVol associated with different measurement locations along the jet needs to be better understood for assessing and potentially improving the reliability of the direct 4D flow CMR-based quantification of RVol. The aim of this study is, therefore, to quantify RVol at multiple locations along the jet to evaluate the impact of measurement location on MR assessment by the direct 4D flow CMR method.

Another aim of this study is to identify the optimal location for the measurement plane along the jet, based on the principle of momentum conservation in a free jet. This principle of fluid dynamics describes the conservation of axial momentum rate in a free jet as it propagates [23]. This principle has been demonstrated in in-vitro pulsatile MR-mimicking jet flow models [22], [24], [25] and has been used to derive an equation for estimating RVol using Doppler echocardiography data [24], [25]. If this conservation of momentum is applicable to in-vivo MR jets, then the momentum profile along the jet derived by 4D flow CMR will be constant except for the signal loss region in which velocities are underestimated. Therefore, the optimal plane location can be identified as the point closest to the regurgitant orifice where the jet momentum converges. This specific location could potentially minimize errors associated with velocity underestimation and prevent overestimation of RVol due to flow entrainment.

## 1.1 Free jet assumption

Free jets are characterized by a fluid flow ejected into a surrounding medium without solid boundaries, allowing the jets to freely expand. In such conditions, jets do not create an external pressure gradient in their surroundings. Therefore, their momentum does not change. MR jets do not exactly resemble a free jet as they are bounded by the wall of the left atrium (LA). However, in the vicinity of the regurgitant orifice, MR jets may exhibit characteristics similar to a free jet, given that the size of the jet is relatively smaller than LA. For instance, the vena contracta width of MR jets is on the order of millimeters (mild MR < 3 mm, severe MR ≥ 7 mm), while LA is on the order of centimeters (normal diameter < 4 cm).

## 2 Materials and methods

### 2.1 Patient recruitment

Adult patients previously diagnosed with MR and referred for CMR were prospectively recruited from July 2019 to September 2020. Inclusion criteria were 1) adults 18 years of age or older, 2) any degree of MR (trivial to severe) documented on echocardiography within 1 year of CMR, and 3) scheduled for a clinically indicated CMR within 3 months of their latest TTE exam. Concomitant valvular diseases were allowed. Exclusion criteria were 1) pregnant and breastfeeding women, 2) diagnosed with persistent or worse atrial fibrillation, and 3) congenital cardiac defects. The study was approved by our institutional review board, and written informed consent was obtained from all participants.

### 2.2 Transthoracic echocardiography

Previously acquired TTE images of patients were examined by two board-certified cardiologists (T.S. and G.A.) to quantify MR severity based on the multi-parametric integrative approach recommended by the American Society of Echocardiography guidelines [26].

### 2.3 Cardiovascular magnetic resonance

Patients underwent a clinically indicated CMR exam for further evaluation of heart conditions. All exams were conducted on 1.5T scanners (Avanto or Aera, Siemens Healthineers, Erlangen, Germany). Balanced steady-state free-precession cine images were acquired for two-, three-, and 4-chamber long-axis and a stack of short-axis to measure LV end-diastolic, end-systolic, and stroke volumes. 2D through-plane velocity-encoded phase-contrast magnetic resonance imaging was acquired at the sinotubular junction to measure aortic forward flow volume. RVol (RVol_indirect_) was calculated by subtracting the aortic forward flow volume from the LV stroke volume. A board-certified cardiologist (T.S.) performed all measurements using commercially available software (cvi42, Circle Cardiovascular Imaging, Calgary, Alberta, Canada).

### 2.4 Four-dimensional flow cardiovascular magnetic resonance

4D flow CMR was acquired at the end of a clinical CMR protocol. A prospective electrocardiogram- and respiratory-gating with generalized autocalibrating partial parallel acquisition acceleration was used. Sagittal oblique field-of-view, covering LV and LA was applied to capture MR. Other sequence parameters were as follows: flip angle = 7°/15° (if contrast agent was admitted), echo time = 2.18–2.52 ms, repetition time = 3.19–5.21 ms, field-of-view = 255–438 × 340–500 × 200–384 mm^3^, acquisition matrix = 110–160 × 160 × 68–96, reconstructed spatial resolution = 3.2–5.2 × 2.1–3.1 × 2.5–4 mm^3^, temporal resolution = 36–42 ms, velocity encoding: 150–250 cm/s. Velocities were corrected for background phase offset and aliasing errors using in-house MATLAB (MathWorks, Natick, Massachusetts) codes based on previous methods by Walker et al. [27]. The 3D LA was segmented using Mimics (Materialise NV, Leuven, Belgium) based on 3D phase-contrast magnetic resonance angiogram generated by averaging the product of 4D flow CMR velocity magnitude and signal magnitude over time. Special care was taken to ensure that the entirety of MR jets was contained within the segmented LA (Fig. S1).

#### 2.4.1 Direct MR quantification method

**Fig. 1:**
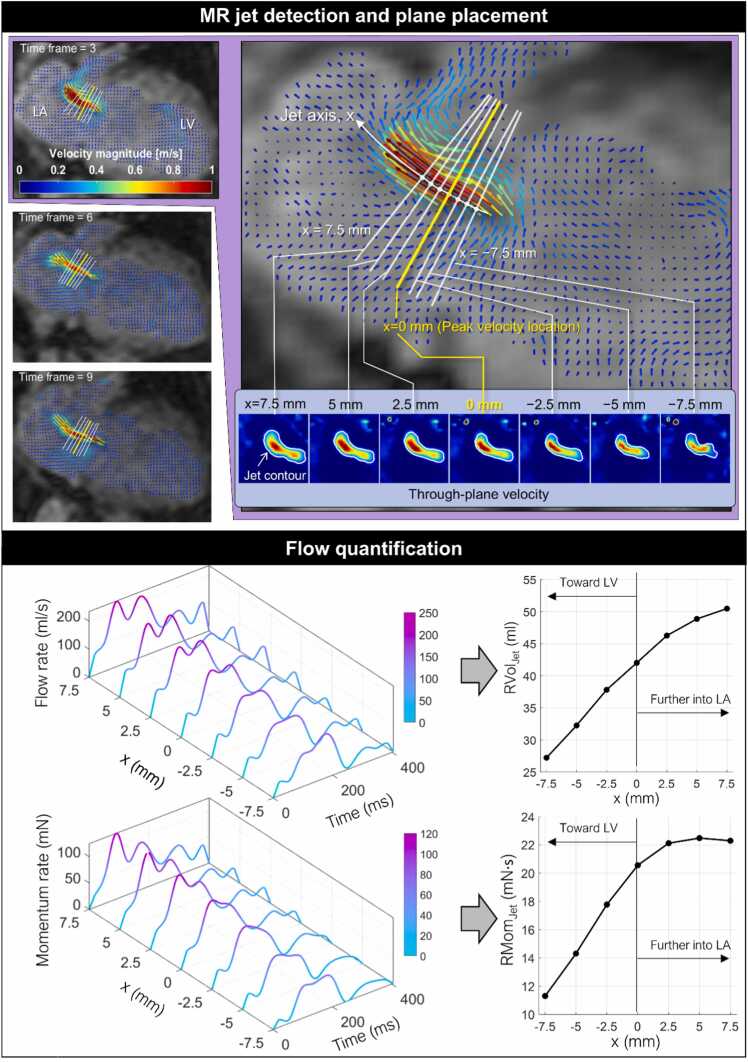
Graphical illustration of measurement plane positioning along the jet (top box) and flow quantification (bottom box). Jet flow rate and momentum rate were quantified at seven distinct locations with an interval of 2.5 mm along the jet axis denoted as x. The reference point (x = 0 mm) corresponds to the location of the voxel containing the highest velocity magnitude within the MR jet at each time point. *MR* mitral regurgitation, *LA* left atrium, *LV* left ventricle, *RVol* regurgitant volume

The flow rate and axial momentum rate of an MR jet were quantified at seven equidistant planes along the jet axis, x, with a 2.5 mm interval (x = −7.5 to 7.5 mm), as illustrated in Fig. 1. The central plane (at x = 0 mm) was positioned at the location of maximum velocity voxel within the MR jet area at each time point, allowing the central plane to dynamically track the movement of the MR jet throughout systole. The entire post-processing, including plane placement and flow quantification, was performed semi-automatically using an in-house MATLAB tool. A detailed description of the quantification process is as follows:

1. The start (t_0_) and end (t_end_) time frames of MR are determined by inspecting LA velocity magnitude and vectors to confirm the presence of a regurgitant jet.
2. At each time point during MR, a voxel with a maximum velocity within the MR jet is identified. This step is performed by automatically searching for a voxel with the maximum velocity within the LA segmentation, followed by manual confirmation that the voxel lies within the jet.
3. The central plane (x = 0 mm) is placed and centered at the maximum velocity voxel. The plane orientation is determined by setting the plane normal vector as the median of the velocity vector at the plane center and its adjacent velocity vectors (i.e., 3 × 3 × 3 median filter), ensuring that the plane is perpendicular to the jet.
4. Three additional planes, spaced 2.5 mm apart, are automatically placed on either side of the central plane along the jet axis. The normal vector of each plane is adjusted using the same approach as in step 3 to ensure each plane is positioned perpendicular to the jet at its respective location.
5. Through-plane velocities at each plane were computed by reformatting the 4D flow CMR velocity vectors onto the plane using linear interpolation with a pixel resolution of 0.5 × 0.5 mm^2^.
6. The jet region is contoured on through-plane velocity images at each plane. Contours were generated automatically using dual-velocity contour propagation. This method first creates an isovelocity contour corresponding to 20% of the plane center velocity, capturing the shape of the jet core. The first contour is then radially dilated until it meets a second isovelocity contour at 0.1 m/s, defining the jet boundary. Manual correction is applied if necessary.

Finally, flow rate and axial momentum rate were computed as follows.

(1) $$Q_{\mathrm{jet}}\left(x\right)=\sum_{i\in \Omega_{\mathrm{jet}}}V_{⊥}^{i}\mathrm{dA}$$

(2) $$M_{\mathrm{jet}}\left(x\right)=\rho \sum_{i\in \Omega_{\mathrm{jet}}}{\mathrm{sgn}\left(V_{⊥}^{i}\right)∙\left(V_{⊥}^{i}\right)}^{2}\mathrm{dA}\;\mathrm{sgn}\left(V_{⊥}^{i}\right)=\;\left\{\begin{array}{c} -1\mathrm{if}V_{⊥}^{i}<0\\ 0\mathrm{if}V_{⊥}^{i}=0\\ 1\mathrm{if}V_{⊥}^{i}>0 \end{array}\right)$$

where $Q_{\mathrm{jet}}$ indicates jet flow rate, $M_{\mathrm{jet}}$ indicates jet axial momentum rate, $V_{⊥}^{i}$ indicates the through-plane velocity at the $i^{\mathrm{th}}$ pixel within the jet area, $\Omega_{\mathrm{jet}}$, dA indicates the pixel area (=0.25 mm^2^), and ρ is the density of blood approximated as the density of water (=1.0 g/cm^3^). The flow rate and momentum rate were integrated over the duration of MR (i.e., from t_0_ to t_end_) to determine regurgitant jet flow volume (RVol_jet_) and regurgitant jet flow momentum (RMom_jet_), respectively. Double-jet MR was noticed in two patients. RVol_jet_ and RMom_jet_ were quantified separately for each jet and then summed.

### 2.5 Inter-observer variability

Two observers (A.A. and J.L.) underwent the MR flow quantification steps 1 through 6 independently in 10 randomly selected patients. RVol_jet_ and RMom_jet_ quantified at all seven planes were compared between the two observers.

### 2.6 Statistical analysis

Continuous variables are reported using mean ± standard deviation. A paired two-tailed t-test or Wilcoxon signed-rank test was used to analyze RVol_jet_ and RMom_jet_ differences between measurement locations along the jet axis. A two-tailed t-test or Wilcoxon rank-sum test was utilized to evaluate differences between two MR severity groups. Bonferroni-adjusted statistical significance level of 0.017 (0.05/3 groups) was used. Bland-Altman analysis was used to evaluate systematic bias and limits of agreement between the direct 4D flow CMR and indirect volumetric CMR methods. Intraclass correlation coefficient (ICC) was used to evaluate inter-modality and inter-observer agreements.

## 3 Results

### 3.1 Study cohort

The final study cohort consisted of 45 patients (age 63 ± 13 years, male n = 26). Baseline characteristics are listed in Table 1. Patients were divided into three groups based on their MR severity: grade 1 (trivial to mild, n = 13), grade 2 (moderate, n = 24), and grade 3+ (worse than moderate, n = 8). MR etiology was functional (n =2 5), primary (n = 12), mixed (n = 9), and post-mitral valve repair surgery (n = 2). The time difference between TTE and CMR was 31 ± 25 days.

**Table 1:** Study cohort characteristics.

Parameters	Value
Age (y)	63±13
Male/female	26/19
Body surface area (m^2^)	1.93±0.24
Cardiac function	
LV end-diastolic volume (mL)	196±70
LV end-systolic volume (mL)	196±70
LV stroke volume (mL)	107±65
LV ejection fraction (%)	88±28
LA volume (mL)*	Minimum 45, maximum 194
LA volume index (mL/m^2^)**	Minimum 26, maximum 101
MR severity	
Grade 1: ≤ Mild	13
Grade 2: Moderate	24
Grade 3+: ≥ Moderate-to-severe	8
MR etiology	
Primary	12
Functional	25
Mixed	9
Post-mitral valve repair	2
Aortic flow***	
Forward flow volume (mL)	75±25
Backward flow volume (mL)	2.1±3.3
Regurgitant fraction (%)	2.9±3.5
Cardiovascular comorbidities	
Dilated cardiomyopathy	17
Hypertrophic cardiomyopathy	9
Mitral valve prolapse	5
Coronary artery disease	1
Atrial septal defect	1
Left atrial mass	1
Left ventricular aneurysm	1
Thoracic aorta aneurysm	1

*2D* two-dimensional, *3D* three-dimensional, *4D* four-dimensional, *CMR* cardiovascular magnetic resonance, *LA* left atrium, *LV* left ventricle, *MR* mitral regurgitation

Data are numbers of cases or means +/- standard deviation.

* The volume of 3D LA region segmented using 4D flow CMR

** Indexed to body surface area

*** Aortic flow parameters derived from aortic valve 2D phase-contrast measurements

### 3.2 RVol_jet_ variation along jet axis

**Fig. 2:**
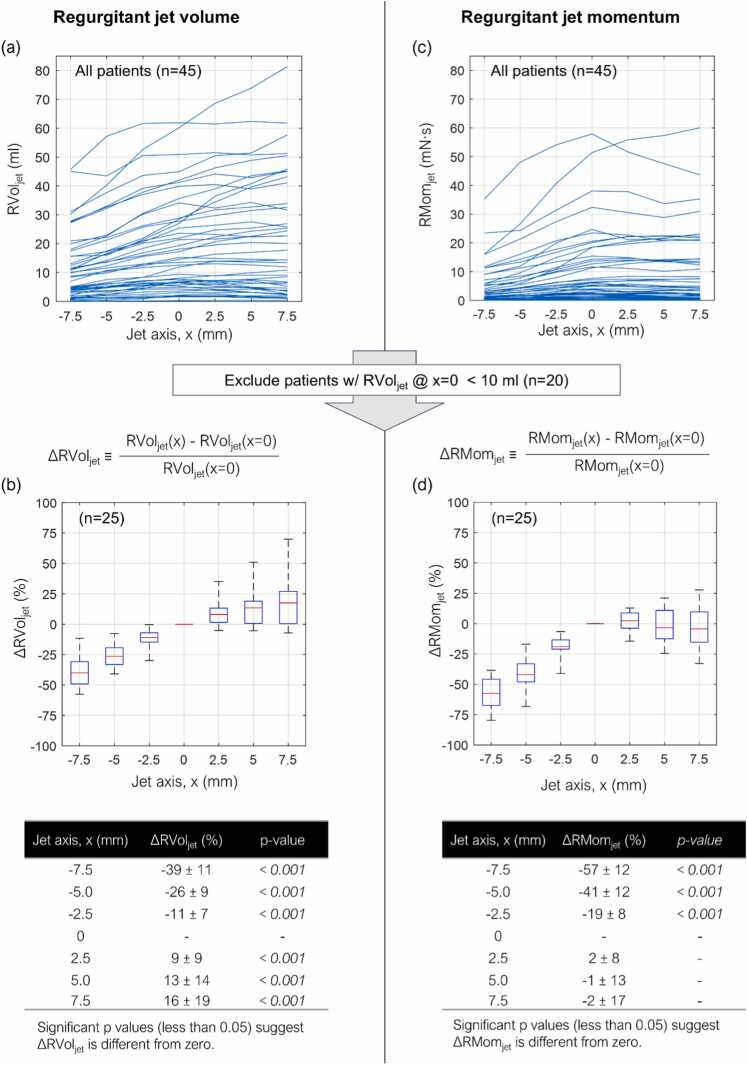
Regurgitant jet volume (RVol_jet_) and regurgitant jet momentum (RMom_jet_) variation along the jet axis. Panels (a) and (c) display the profiles of RVol_jet_ and RMom_jet_, respectively, while panels (b) and (d) depict their relative changes with respect to the peak velocity location (x = 0 mm). Note that relative changes are analyzed in a subset of 25 patients with RVol_jet_ ≥ 10 mL at x = 0 mm

There was a continuous increase in RVol_jet_ further into LA (Fig. 2a). The percentage change in RVol_jet_ relative to the measurement at the peak velocity location was computed in patients with non-trivial MR, defined as RVol_jet_ ≥ 10 mL at the peak velocity location. Compared to RVol_jet_ measured at the peak velocity location, RVol_jet_ showed 39 ± 11% (p < 0.001) reduction at the location closest to LV, i.e., x = −7.5 mm, and 16 ± 19% (p < 0.001) increment at the furthest downstream into LA, i.e., x = 7.5 mm (Fig. 2b). MR severity was re-classified using RVol_jet_ measured at different locations using the RVol thresholds: MR 1 (RVol < 30 mL), MR 2 (30 mL ≤ RVol < 45 mL), and MR 3+ (RVol > 45 mL). Due to the continuous increase in RVol_jet_, the number of patients with MR 1 reduced from 41 (91%) to 32 (71%) and patients with MR 3+ increased from 2 (4%) to 8 (18%), as the measurement location moved further into LA from x = −7.5 mm to x = 7.5 mm (Table 2).

**Table 2:** Mitral regurgitation (MR) severity classified by RVol_jet_ obtained at different locations along the jet axis.

Jet axis, x (mm)	MR 1	MR 2	MR 3+
−7.5 (closest to LV)	41 (91%)	2 (4%)	2 (4%)
−5.0	38 (84%)	6 (13%)	1 (2%)
−2.5	36 (80%)	6 (13%)	3 (7%)
0 (at peak jet velocity)	36 (80%)	6 (13%)	3 (7%)
2.5	33 (73%)	7 (16%)	5 (11%)
5.0	32 (71%)	8 (18%)	5 (11%)
7.5 (furthest into LA)	32 (71%)	5 (11%)	8 (18%)

The following clinically accepted RVol thresholds were applied: RVol ≤ 30 mL (MR 1), 30 < RVol ≤ 45 mL (MR 2), and 45 < RVol (MR 3+)

*LA* left atrium, *LV* left ventricle, *RVol* regurgitant volume

Data are numbers of cases or means +/- standard deviation.

### 3.3 RMom_jet_ variation along jet axis

There was a continuous increase in RMom_jet_ further into LA until the peak velocity location, x = 0 mm (Fig. 2c). They remained relatively stable after the peak velocity location compared to RVol_jet_. The percentage change in RMom_jet_ was computed in a similar manner to RVol_jet_. RMom_jet_ showed 57 ± 12% (p < 0.001) reduction at the location closest to LV, i.e., x = −7.5 mm, which slowly recovered toward the peak velocity location. After the peak velocity location, RMom_jet_ remained statistically unchanged (Fig. 2d).

### 3.4 RVol agreement between two techniques: RVol_jet_ and RVol_indirect_

**Fig. 3:**
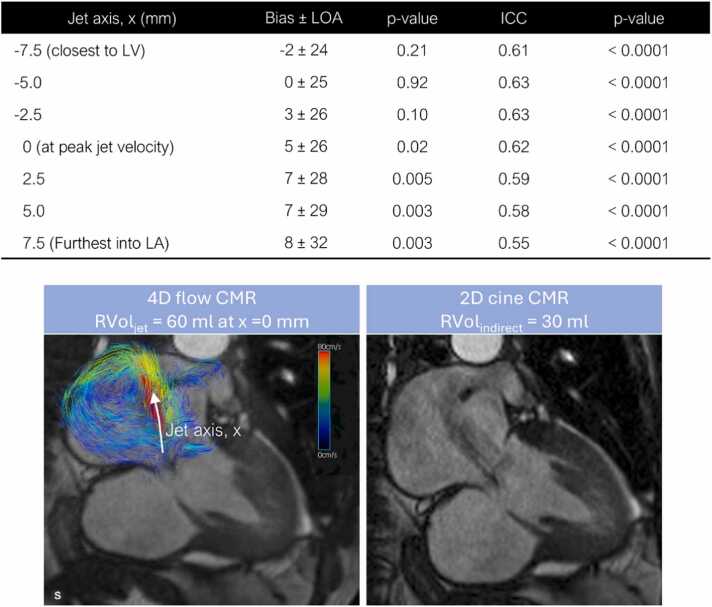
Comparison between the indirect CMR-derived RVol (RVol_indirect_) and the direct 4D flow CMR-derived RVol (RVol_jet_) quantified at various locations along the jet. Bland-Altman analysis results, including mean bias and limits of agreement (LOA), along with intraclass correlation coefficient (ICC) values, are summarized in the table. Example 4D flow (lower left) and 2D cine CMR (lower right) images from a patient with severe functional mitral regurgitation with a left ventricular basal aneurysm are shown below the table. Note that the 4D flow CMR image depicts blood flow pathlines in the left atrium derived from 4D flow CMR, with a two-chamber cine image embedded in the background to provide anatomical context. *2D* two-dimensional, *4D* four-dimensional, *CMR* cardiovascular magnetic resonance, *LA* left atrium, *LV* left ventricle, *RVol* regurgitant volume

RVol_indirect_ was obtained in 41 out of 45 patients due to missing 2D aortic flow phase-contrast images in the 4 initial patients. Of those 41 patients, no significant aortic regurgitation was noted based on the regurgitation fraction measured by the 2D aortic phase contrast as shown in Table 1. There was moderate agreement (ICC = 0.55–0.63, p < 0.001) between RVol_jet_ and RVol_indirect_ at all seven measurement locations (Fig. 3). The mean difference (RVol_jet_ minus RVol_indirect_) increased from −2 ± 24 mL to 8 ± 32 mL, as the measurement location moved into LA. Correlation and Bland-Altman plots between RVol_jet_ and RVol_indirect_ at all seven locations are included as Supplementary Information (Fig. S2).

### 3.5 Relationship with MR severity

**Fig. 4:**
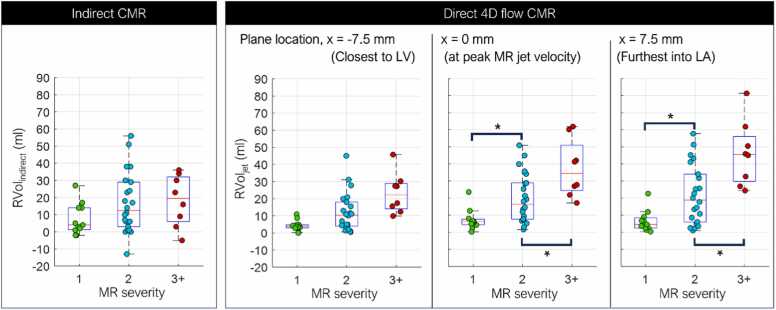
Distribution of indirect CMR-derived RVol and direct 4D flow CMR-derived RVol across MR severity groups. The asterisk symbol (*) indicates a significant difference in RVol between two groups, with a p-value less than 0.017, corresponding to the Bonferroni-corrected alpha level of 0.05. *4D* four-dimensional, *CMR* cardiovascular magnetic resonance, *MR* mitral regurgitation, *RVol* regurgitant volume

Fig. 4 shows the distribution of RVol_indirect_ and RVol_jet_ in TTE-based MR severity 1, 2, and 3+ groups. RVol_indirect_ (8 ± 9 vs 19 ± 18 vs 18 ± 15 mL) and RVol_jet_ measured closest to LV (4 ± 3 vs 12 ± 11 vs 23 ± 12 mL) were not statistically different between all groups. RVol_jet_ measured at the peak velocity location (7 ± 6 vs 20 ± 14 vs 37 ± 17 mL) and the furthest into LA (7 ± 6 vs 22 ± 17 vs 46 ± 19 mL) demonstrated significant increase with increasing MR severity.

### 3.6 Inter-observer reproducibility

RVol_jet_ (ICC = 0.988, bias = −1 ± 5.2 mL) and RMom_jet_ (ICC = 0.997, bias = −0.2 ± 2.8 mN·s) quantified by the two observers demonstrated excellent agreement.

## 4 Discussion

This study demonstrates how direct quantification of MR RVol using 4D flow CMR can be affected by the choice of a measurement location along the jet. We quantified the profile of RVol_jet_ along the jet direction within ±7.5 mm distance from the peak MR jet velocity location. We found that RVol_jet_ continuously increases as the measurement location moves further into LA. The degree of variability was large enough to alter MR severity classified based on RVol_jet_. In addition, the momentum profile of the MR jet (RMom_jet_) quantified using 4D flow CMR revealed the presence of severe velocity underestimation in the region upstream of the peak velocity. These findings suggest the peak velocity location as the optimal measurement plane location to directly quantify RVol using 4D flow CMR.

In previous studies, a common strategy for measuring plane positioning was to place a plane slightly distal to the regurgitant orifice to avoid severe signal loss occurring in the vicinity of the orifice [16], [17], [18], [19], [20], [21]. A measurement plane located at the regurgitant orifice has been shown to significantly underestimate RVol compared to using a plane 1–2 cm above the regurgitant orifice [17]. Severe signal loss in the vicinity of the orifice/stenosis is a common observation in phase-contrast images of jets [28], [29], [30]. This local signal loss has been shown to be caused by a wide intravoxel phase dispersion, which is triggered by drastic changes in the direction and magnitude of blood velocities (i.e., high convective acceleration) as the flow navigates through a constriction. Such a phenomenon has been demonstrated to result in velocity underestimation in phase-contrast images [28], [30]. In light of these previous findings, our observation of the progressive reduction in RVol_jet_ as the measurement plane moves toward LV may be due to the increasing degree of velocity underestimation, dominating over the flow rate increase caused by the flow entrainment.

Our in-vivo observation of the converging pattern of the RMom_jet_ profile along the jet offers valuable insight into the extent of a jet where near-orifice velocity underestimation impacts flow quantification using 4D flow CMR. Stabilization of RMom_jet_ as the measurement plane moves beyond the peak velocity location suggests that the influence of velocity underestimation may have diminished at the peak velocity location. This may also explain in part why the peak velocity location appears slightly distal to the mitral valve. Beyond the peak velocity point, RVol overestimation due to flow entrainment may become the primary source of error, as shown by the continuous increase in RVol_jet_ in Fig. 2a. Therefore, positioning a measurement plane at the peak velocity location may provide the most optimal estimate of RVol using the direct 4D flow CMR approach. In addition, having the peak velocity set as a hemodynamic landmark to place a plane could improve the consistency and reproducibility of RVol measurements. A prior study by Gupta et al. utilized peak velocity as a reference point to position a measurement plane and reported that the direct 4D flow CMR method achieved higher inter-observer reproducibility compared to the indirect volumetric CMR method (ICC = 0.97 vs 0.80) in hypertrophic cardiomyopathy patients [20].

RVol_jet_ demonstrated moderate agreement with RVol_indirect_ across all measurement locations (ICC = 0.55–0.63, mean bias = −2 to 8 mL) in the present study. While altering the MR measurement plane location did not significantly affect the agreement level, a small displacement of the measurement plane within a ±7.5 mm range did affect the mean differences between the two techniques. This dependency on measurement location may partly explain the mixed findings of underestimation (−5 ± 16 mL [18] and −6 ± 25 mL [20]) and overestimation (11 ± 43 mL [16] and 6 ± 31 mL [17]) of RVol by direct 4D flow CMR compared to indirect volumetric assessment. It may also help explain why the limits of agreement remain wide, as observed in this study (±24 to ±32 mL) and in previous reports [16], [17], [18], [20], [31]. The considerable variability in agreement may also reflect inherent limitations in each technique.

The indirect volumetric method carries a risk of error amplification, as combining multiple volume measurements of similar magnitude amplifies the intrinsic uncertainty of each measurement, increasing the overall uncertainty. For instance, if the LV end-diastolic and end-systolic volumes and aortic forward flow volume each have an uncertainty of ±10 mL, the combined uncertainty when they are subtracted increases by a factor of the square root of 3, resulting in approximately ±17 mL. Additionally, complex LV and mitral valve structures may impact RVol quantification using the indirect volumetric approach. In hypertrophic cardiomyopathy patients, the inclusion or exclusion of hypertrophied trabeculations and papillary muscles as part of the blood pool has been shown to significantly alter LV stroke volume and consequently, RVol [13], [32]. Additionally, Gupta et al. [20] reported LV end-systolic volume has only moderate inter-observer reproducibility (ICC = 0.69), while LV end-diastolic volume and aortic forward flow volume have excellent reproducibility (ICC > 0.9) in hypertrophic cardiomyopathy patients. In patients with mitral valve prolapse, an additional LV cavity is created beneath the mitral valve as the leaflets bulge toward the left atrium. The exclusion of this “prolapse volume” has been shown to estimate significantly higher RVol compared to when the prolapse volume is considered as part of LV end-systolic volume (31 ± 20 mL vs 14 ± 19 mL [14], 37 ± 23 mL vs 21 ± 24 mL [15]). In the present study, mitral valve prolapse was observed in five patients and hypertrophic cardiomyopathy in nine patients. Across all measurement plane locations, both subgroups demonstrated wide limits of agreement ranging from ±20 to ± 38 mL between the direct 4D flow CMR and indirect CMR assessments, while the mean bias was less than 8 mL. However, the small sample sizes in these subgroups limited the ability to draw statistically meaningful observations.

On the other hand, the direct 4D flow CMR method carries potential errors arising from measurement location dependency, as demonstrated in this study, as well as inadequate voxel coverage of the MR jet [22]. Consistent with the findings of the present study, Blanken et al. [17]. demonstrated that the valve tracking approach, which typically places the measurement plane at the mitral valve level, results in significantly lower RVol (10 [6–17] mL vs 25 [14, 46] mL) compared to flow tracking, which positions the measurement plane 1–2 cm above the mitral valve. The number of voxels across the jet cross-section could influence the accuracy of direct jet flow quantification, as demonstrated in an in-vitro study by Lee et al. [22]. They reported a 5% underestimation in jet flow volume quantification when four voxels covered the jet's cross-sectional area, with fewer voxels leading to greater underestimation. Assuming a typical 4D flow CMR has an isotropic voxel size of 2.5 mm, four voxel coverage would require an MR jet with a cross-sectional diameter of 1 cm. Such a jet would have a cross-sectional area of 0.79 cm^2^, which exceeds the regurgitant orifice area threshold for severe MR (0.4 cm^2^). Our 4D flow CMR data consisted of voxels larger than 2.5 mm, suggesting that the RVol_jet_ might be smaller than true RVol in certain patients with small MR jets (e.g., mild MR). Overall, this study does not provide evidence that the direct 4D flow CMR technique is more accurate than conventional methods, which is challenging due to the absence of a gold-standard quantification technique. Instead, it highlights the internal variability of the direct 4D flow CMR approach associated with measurement location, and our efforts to address this issue by examining the momentum profile of the MR jet.

## 5 Limitations

This study has certain limitations. The cohort in our study exhibited diverse mechanisms of MR, including functional MR (n = 25), primary MR (n = 12), a combination of functional and primary MR (n = 9), and post-mitral valve repair (n = 2). Functional MR resulting from a dilated mitral annulus or primary MR caused by bileaflet mitral valve prolapse tends to produce centrally directed MR jets. On the other hand, functional MR induced by systolic anterior motion of the mitral valve leaflet associated with hypertrophic cardiomyopathy or primary MR caused by flail leaflet or single leaflet prolapse tends to produce eccentric jets. Our study cohort indeed comprised a mixture of both central and eccentric jets. Compared to central jets, eccentric jets will entrain less amount of fluid, and their momentum will no longer remain conserved as these jets are often attached to the proximal mitral valve leaflet or the left atrial wall. The close proximity to a wall results in a restrictive entrainment of surrounding fluid and loss of momentum due to wall friction [33]. This could account for the observed increase in variability of relative changes in RVol_jet_ and RMom_jet_ when the measurement plane moves further into LA from the peak velocity location (Fig. 2b and d). Further investigation is warranted to evaluate relationships between flow eccentricity and the robustness of the direct 4D flow CMR quantification approach. Nonetheless, the consistent decrease in both RVol_jet_ and RMom_jet_ toward LV from the peak velocity location confirms the dominance of velocity underestimation in the region. Second, although we showed excellent inter-observer reproducibility of the direct 4D flow CMR quantification of MR flow parameters, manual 3D LA segmentation was performed only once and shared by the two observers. Potential variation in segmentation quality near the regurgitant orifice could introduce variability in peak velocity location detection. Future development of automated 3D LA segmentation would further improve the reliability and clinical utility of the direct 4D flow CMR quantification technique employed in this study.

## 6 Conclusion

The direct RVol quantification in MR patients using 4D flow CMR is heavily influenced by the choice of measurement location along the MR jet. RVol estimation variability introduced by measurement location variability could impact MR severity classification. This underscores the necessity for a robust criterion for locating the measurement plane along the jet. Guided by the momentum conservation principle, we propose that the location of peak velocity of the MR jet as an optimal measurement location to estimate RVol using direct 4D flow CMR approach.

## Author contributions

Gloria Ayuba: Writing—review and editing, Validation, Formal analysis. Vinesh Appadurai: Writing—review and editing, Validation, Methodology, Formal analysis. Adarsh Aratikatla: Writing—original draft, Visualization, Methodology, Formal analysis. Taimur Safder: Writing—review and editing, Validation, Investigation, Formal analysis. Michael Markl: Writing—review and editing, Supervision, Resources. Jeesoo Lee: Writing—review and editing, Writing—original draft, Visualization, Validation, Supervision, Project administration, Methodology, Investigation, Funding acquisition, Formal analysis, Data curation, Conceptualization. Aakash Gupta: Writing—review and editing, Resources, Methodology, Data curation. James Thomas: Writing—review and editing, Supervision, Resources, Methodology, Conceptualization.

## Declaration of generative AI and AI-assisted technologies in the writing process

During the preparation of this work, the authors used ChatGPT solely to enhance readability and correct grammatical errors. After using this tool/service, the authors reviewed and edited the content as needed, and take full responsibility for the content of the publication.

## Declaration of competing interests

The authors declare that they have no known competing financial interests or personal relationships that could have appeared to influence the work reported in this paper.

## Data Availability

The datasets generated and/or analyzed during the current study are available from the corresponding author upon reasonable request.
