# Supplementary material for: Impact of measurement location on direct mitral regurgitation quantification using four-dimensional flow cardiovascular magnetic resonance
Source: J Cardiovasc Magn Reson. 2025 Jan 26;27(1):101847. doi: 10.1016/j.jocmr.2025.101847 (PMC11870250; doi:10.1016/j.jocmr.2025.101847)
Supplement: Supplementary file 1 — Supplementary material [file mmc1.docx]

**
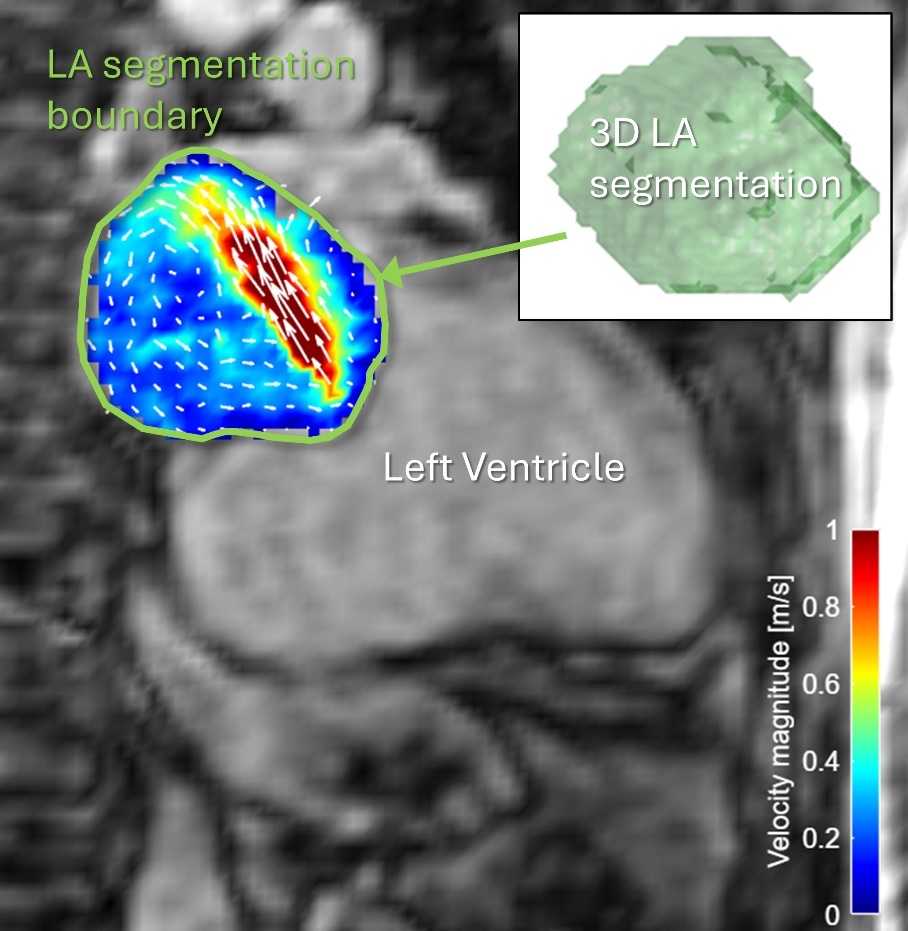
**

**Figure S1. Example of 3D left atrial (LA) segmentation.** The 3D LA boundary is overlaid on both 4D flow CMR magnitude and segmented velocity magnitude, with an inset displaying the 3D shape of the LA segmentation. Special attention was given to ensure that the mitral regurgitant jet visible on 3D phase-contrast magnetic resonance angiogram is captured in the segmentation. The left atrial appendage and pulmonary veins were generally excluded to simplify the segmentation process. This exclusion did not affect our ability to detect the peak velocity of the mitral regurgitant jet, which forms near the mitral valve.


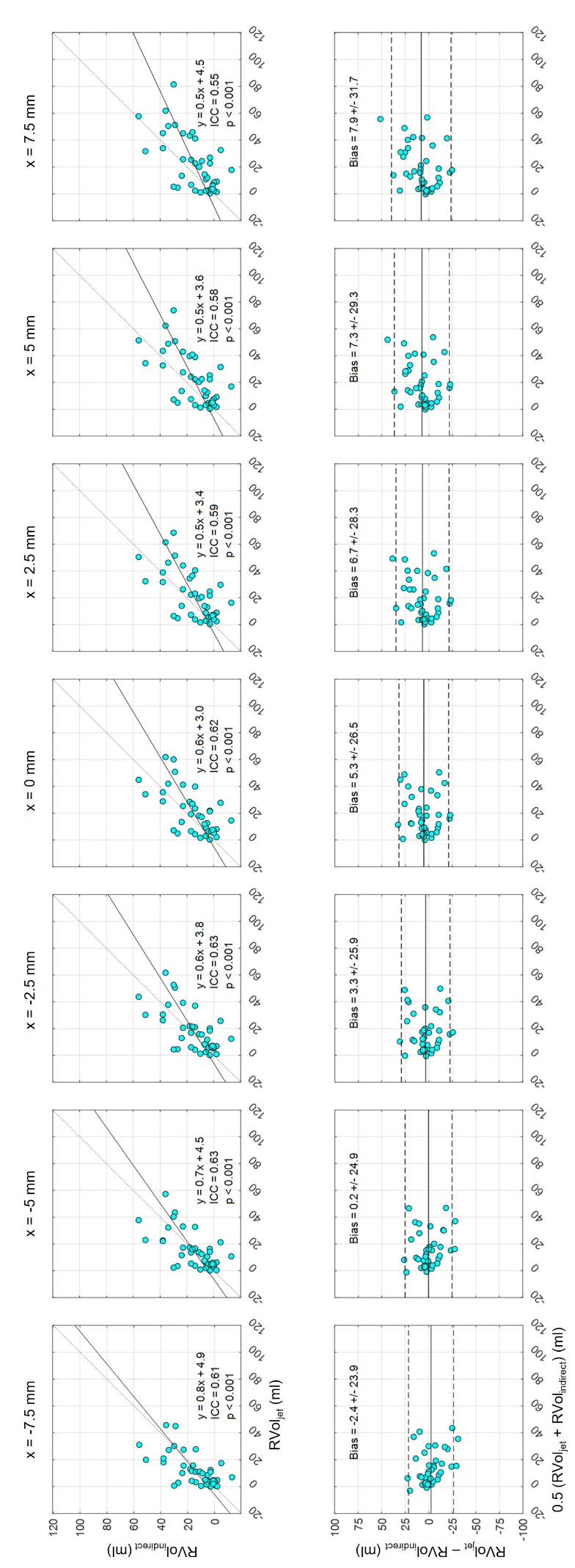


**Figure S2.** Correlation and Bland-Altman plots between RVol_indirect_ and RVol_jet_ at all measurement locations along the jet axis
